# A Novel HDAC6 Inhibitor Ameliorates Imiquimod-Induced Psoriasis-Like Inflammation in Mice

**DOI:** 10.3390/molecules30153224

**Published:** 2025-07-31

**Authors:** Anqi Cao, Yurong Li, Yanqiao Feng, Xiaoquan Wang, Wenyu Wei, Hongyan Sun, Junmin Quan

**Affiliations:** 1 State Key Laboratory of Chemical Oncogenomics, Guangdong Key Laboratory of Chemical Genomics, Peking University Shenzhen Graduate School, Shenzhen 518055, China; 2 Inno Biopharmaceuticals (Shenzhen) Co., Ltd., Shenzhen 518055, China; 3 State Key Laboratory of Efficient Utilization of Arable Land in China, the Institute of Agricultural Resources and Regional Planning, Chinese Academy of Agricultural Sciences, Beijing 100081, China; 4 Department of Chemistry and Center of Super-Diamond and Advanced Films (COSDAF), City University of Hong Kong, 83 Tat Chee Avenue, Kowloon, Hong Kong 999077, China

**Keywords:** psoriasis, inflammation, HDAC6, MAPK, STAT3

## Abstract

Psoriasis is a chronic inflammatory skin disease characterized by abnormal proliferation of keratinocytes and infiltration of inflammatory cells. Significant challenges remain in developing effective and safe targeted therapies for psoriasis. Here, we reported the discovery of novel cystamine derivatives for the treatment of psoriasis. These compounds effectively attenuated LPS-induced inflammation in vitro, and the optimal candidate CS1 ameliorated imiquimod-induced psoriasis-like inflammation in mice. Mechanistically, CS1 bound and inhibited the deacetylase HDAC6, subsequently inhibited the AKT, MAPK, and STAT3 pathways, attenuated the hyperproliferation and altered differentiation of keratinocytes and reduced the infiltration of immune cells. These findings suggest that HDAC6 may serve as a potential target for drug development in the treatment of psoriasis.

## 1. Introduction

Psoriasis is a complex immune-mediated hyperproliferative skin disorder that affects approximately 2% of the global population, which is characterized by abnormal terminal differentiation of keratinocytes and significant infiltration of immune cells [1]. Despite extensive researches, the precise mechanisms underlying the development and progression of psoriasis remain incompletely understood. Nonetheless, it is widely accepted that aberrant activation of immune cells mainly contributes to the onset and progression of this condition [2]. The increasing release of inflammatory cytokines, including IL-6, IL-17A, IL-22, IL-23, and TNF-a, by immune cells causes the worsening of epidermal symptoms and ultimately aggravating the psoriasis condition [3]. Psoriasis is often associated with many autoinflammatory comorbidities, including psoriatic arthritis, vitiligo, autoimmune thyroiditis, rheumatoid arthritis, and inflammatory bowel disease (IBD), resulting in a substantial impact on the quality of life of patients [4,5].

Based on the severity of the disease, various pharmaceutical agents are employed in clinical practice [6]. Patients with mild to moderate symptoms typically receive topical medications, such as corticosteroids and calcineurin inhibitors, or novel small molecule drugs like JAK inhibitors, PDE4 inhibitors, and AhR agonists. Systemic therapies including biological agents and some oral low MW agents are applied for the treatment of moderate-to-severe plaque psoriasis. Currently, biological agents, such as TNF-a inhibitors and antibodies against IL12/23 and IL17, are recommended as the first-line treatments for moderate-to-severe psoriasis, but some drawbacks including high cost, side effects, and drug resistance limit their applications. Moreover, low MW agents are also limited by the relatively low efficacy and diverse side effects, suggesting an unmet medical need for the treatment of psoriasis [7,8,9]. Given that almost all these agents have immunomodulatory and anti-inflammatory effects, the development of novel anti-inflammatory agents with improved efficacy and safety profiles is thus highly imperative.

Cysteamine and its oxidized form, cystamine, are endogenous aminothiols produced from coenzyme A metabolism catalyzed by pantetheinase vanin-1, which play a key role in oxidative stress and inflammation [10]. Cysteamine has been approved for more than four decades for the treatment of cystinosis, a lysosomal storage disorder caused by mutations in *CTNS* gene, in which cysteamine mainly functions through it cystine-depleting effect and antioxidative properties. Increasing evidences support the beneficial properties of cysteamine and cystamine in various diseases such as nonalcoholic fatty liver disease (NAFLD) and neurodegenerative and neuropsychiatric disorders [11,12]. We recently showed that a cystamine derivative MC001 effectively suppressed neuroinflammation and alleviated motor deficits in the MPTP-model of Parkinson’s disease [11,12]. In this study, we reported the discovery of novel cystamine derivatives for the treatment of psoriasis. These compounds effectively attenuated LPS-induced inflammation in vitro, and the optimal candidate CS1 ameliorated imiquimod-induced psoriasis-like inflammation in mice.

## 2. Results

### 2.1. Cystamine-Based Compounds Effectively Inhibit LPS-Induced Inflammation

**Figure 1:**
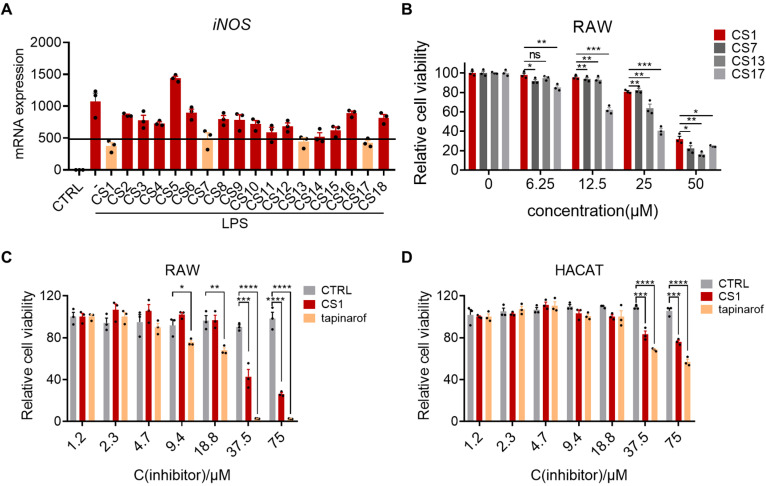
Cystamine-based compounds effectively inhibit LPS-induced inflammation. (**A**) mRNA levels of iNOS in RAW264.7 cells were examined by qPCR. (**B**) CCK8 assay was performed to determine the cell viability of RAW264.6 cells treated with 4 hits. (**C**,**D**) CCK8 assays were performed to determine the cell viability of RAW264.7 and HACAT cells incubated with CS1 for 24 h. The values are presented as the mean ± SEM (n = 3 independent samples). * *p* < 0.05, ** *p* < 0.01, *** *p* < 0.001, and **** *p* < 0.0001 vs. CS1 (**B**) or CTRL (**C**,**D**). *p*-values were calculated using ordinary one-way ANOVA (**B**) and unpaired two-tailed Student’s *t*-test (**C**,**D**).

To improve the lipophilicity of cystamine for skin penetration, a series of hydrophobic derivatives of cystamine were synthesized (Table S1) and further screened in an LPS-induced cell inflammation model. Given the pivotal role of macrophages in the pathophysiology of psoriasis [13,14,15], we established a cellular inflammation model using murine macrophage RAW264.7 cells treated with LPS, in which iNOS was used as the inflammatory marker. Subsequently, a total of 18 synthesized cystamine derivatives were tested at a concentration of 10 mM, and 4 hits (CS1, CS7, CS13, and CS17) were selected for further characterization at a cut-off of 55% inhibition. The cytotoxic effects of the 4 hits were assessed in RAW264.7 cells using the CCK8 assay, and CS1 exhibited less cytotoxicity compared to the other 3 hits (Figure 1B). Given the better anti-inflammatory activity and less cytotoxicity, CS1 was selected for further evaluation. We next determined the effect of CS1 on the viability of macrophages and keratinocytes by CCK8 assay, and the newly approved drug Tapinarof was used as the positive control. The IC50 values of CS1 in RAW264.7 and HaCaT cells were 28.4 μM and 435 μM, respectively, while the IC50 values of Tapinarof in RAW264.7 and HaCaT cells were 8.8 μM and 88.17 μM, respectively (Figure 1C,D), suggesting that CS1 is less toxic than Tapinarof.

### 2.2. CS1 Effectively Inhibits LPS-Induced Inflammation in RAW264.7 Cells

**Figure 2:**
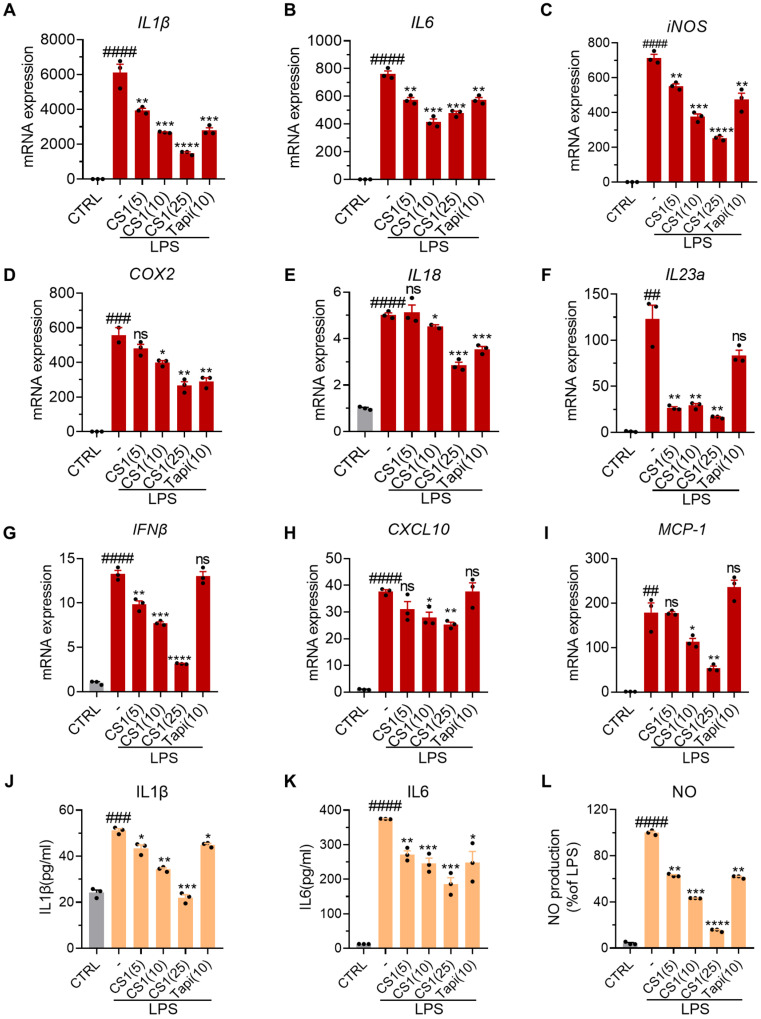
CS1 suppresses inflammatory stimuli-mediated cytokines and chemokine levels. (**A**–**I**) mRNA levels of inflammatory cytokines in RAW264.7 cells were examined by qPCR. (**J**–**L**) The levels of IL1β, IL6, and NO in the culture supernatant of RAW cells. The values are presented as the mean ± SEM (n = 3 independent samples). ## *p* < 0.01, ### *p* < 0.001, and #### *p* < 0.0001 vs. CTRL. * *p* < 0.05, ** *p* < 0.01, *** *p* < 0.001, and **** *p* < 0.0001 vs. LPS. *p*-values were calculated using ordinary one-way ANOVA.

To further characterize the anti-inflammatory activity of CS1, we employed real-time quantitative PCR (qPCR) to examine mRNA levels of various inflammatory cytokines and mediators in RAW264.7 induced by LPS in the absence or presence of CS1, and Tapinarof was used as the positive control (Figure 2). LPS treatment markedly induced the expression levels of *Il-1β*, *Il-6*, *iNOS*, *Cox2*, *Il-18*, *Il23a*, *Ifn-b*, *Cxcl10*, and monocyte chemoattractant protein-1 (*MCP-1*), while the mRNA levels of all these cytokines or mediators were significantly suppressed by CS1 in a dose-dependent manner. Notably, the positive control Tapinarof inhibited the mRNA levels of some genes, such as *Il-1β*, *Il-6*, *iNOS*, *Cox2*, and *Il-18*, but did not significantly suppress the expression of *Il23a*, *Ifn-b*, *Cxcl10*, and *MCP-1*, suggesting that CS1 has a broader anti-inflammatory effect than Tapinarof. Consistently, CS1 treatment significantly inhibited the production of Il-1β, Il-6, and NO in RAW264.7 cells induced by LPS.

### 2.3. CS1 Attenuates IMQ-Induced Psoriasis-Like Phenotype in Mice

**Figure 3:**
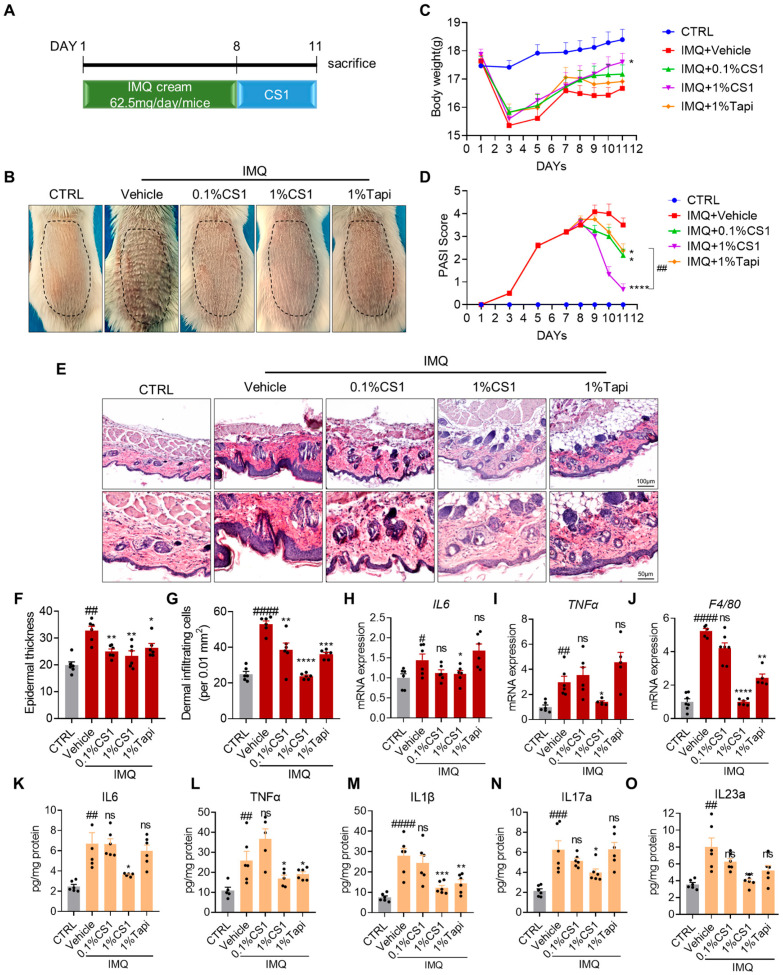
CS1 intervention attenuates Imiquimod (IMQ)-induced psoriasis in Balb/c mice. (**A**) Mice were subjected to a daily dose of 62.5 mg of IMQ cream on the shaved back skin for one week. From day 8, mice were treated with PEG400 or CS1 for 3 days. At the end of the experiment, all mice were sacrificed for following assay. (**B**) Mice were photographed, and the representative photos were presented. (**C**) Mice were weighted every two days during days 1–7 and weighted daily during days 8–11. (**D**) The psoriasis area and severity index (PASI) score was recorded every two days during days 1–7 and calculated daily during days 8–11. (**E**) Hematoxylin and eosin (H&E) staining of mice back skin. (**F**,**G**) The epidermal thickness and dermal infiltrating cells were measured by Image J software. (**H**–**J**) mRNA levels of inflammatory cytokines in mice back skins were examined by qPCR. (**K**–**O**) The levels of cytokines and chemokines in the skin tissues. The values are presented as the mean ± SEM (n = 6 mice per groups). # *p* < 0.05, ## *p* < 0.01, ### *p* < 0.001, and #### *p* < 0.0001 vs. CTRL. * *p* < 0.05, ** *p* < 0.01, *** *p* < 0.001, and **** *p* < 0.0001 vs. vehicle. *p*-values were calculated using unpaired two-tailed Student’s *t*-test (**C**,**D**) and ordinary one-way ANOVA (**F**–**O**).

To assess whether CS1 treatment alleviates IMQ-induced psoriasis-like skin inflammation in mice, we applied IMQ cream topically on the shaved back skin of mice for seven consecutive days, mice were then treated with vehicle, 0.1% and 1% CS1, and 1% Tapinarof (the positive control) for three days (Figure 3A). IMQ topical application profoundly increased erythema, scaling, and thickening of the skin in mice compared with the control group, whereas all treated groups showed a notable decrease in psoriasis-like features and the psoriasis area and severity index (PASI) score, and 1% CS1 exhibited better efficacy compared with 1% Tapinarof (Figure 3B,D). Furthermore, the IMQ application markedly induced body weight loss compared to the control group. While all treated group showed the tendency to attenuate body weight loss, only 1% CS1 significantly rescued body weight loss. Furthermore, histological analysis of the skin tissue showed that IMQ application induces epidermal hyperplasia (acanthosis), parakeratosis in the epidermis, and dermal hyperkeratosis, while the epidermal thickness as well as immune infiltration was markedly reduced by the treatment of CS1 and Tapinarof (Figure 3E–G). Meanwhile, CS1 and Tapinarof exhibited no liver and kidney toxicity as reflected by the levels of ALT, AST, and BUN (Figure S1).

We next evaluated the chemokines and cytokines in the back skin of mice by qPCR and ELISA assays. qPCR results indicated that topical IMQ markedly induced the expression of inflammatory markers including *Il-6*, *Tnf-a*, and *F4/80*, which was significantly suppressed by 1% CS1. Moreover, ELISA assays showed that IMQ resulted in a notable increase in the production of pro-inflammatory cytokines such as IL6, TNF-α, IL-1β, IL-17, and IL-23, while the administration of 1% CS1 effectively inhibited the production of these cytokines (Figure 3H–O). Collectively, the results indicate that CS1 effectively ameliorates IMQ-induced psoriasis-like inflammation and skin lesion in mice.

### 2.4. CS1 Inhibits the Infiltration of Immune Cells

**Figure 4:**
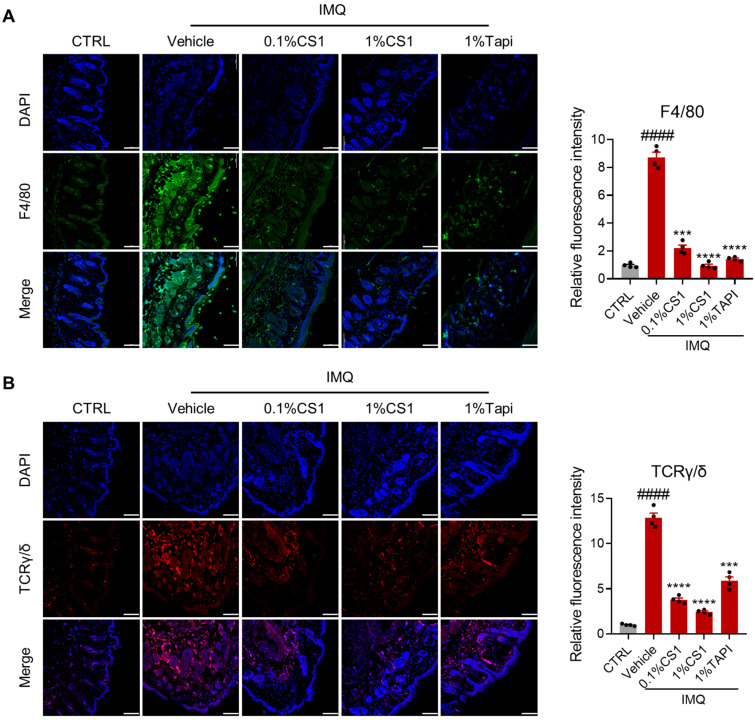
CS1 reduces infiltration of macrophages and TCRγ/δ cells in skin. (**A**) Skin sections were stained with antibodies against F4/80 (green) and DAPI (blue). (**B**) Skin sections were stained with antibodies against TCRγ/δ (red) and DAPI (blue). Scale bar: 100 μm. The values are presented as the mean ± SEM (n = 4 mice skin samples per groups). #### *p* < 0.0001 vs. CTRL. *** *p* < 0.001 and **** *p* < 0.0001 vs. Vehicle. *p*-values were calculated using ordinary one-way ANOVA.

Macrophages serve as the primary cellular component responsible for initiating inflammatory responses within psoriasis lesions [13]. These cells exhibit a significant capacity to infiltrate the dermal layer of psoriatic skin lesions [16]. Therefore, we examined the effect of CS1 on macrophage infiltration in the dermis of psoriasis lesions in mice. Immunofluorescence analysis revealed that IMQ considerably enhanced the infiltration of F4/80-positive macrophages in the skin of mice, which was significantly inhibited by CS1 in a dose-dependent manner (Figure 4A). Consistent with the previous study [17,18], the positive control Tapinarof also significantly reduced macrophage infiltration. Furthermore, CS1 treatment significantly suppressed the infiltration of gamma delta T cells (γδ T cells) in skin lesion induced by IMQ (Figure 4B). Given that γδ T cells are the major IL-17-producing cells in the skin in response to IL-23 stimulation [19,20,21,22], the inhibition of γδ T cells accumulation in skin lesion by CS1 may account for its suppression of the production of IL-17a in skin tissue of mice treated with IMQ.

### 2.5. CS1 Inhibits the Hyperproliferation and Altered Differentiation of Keratinocytes

**Figure 5:**
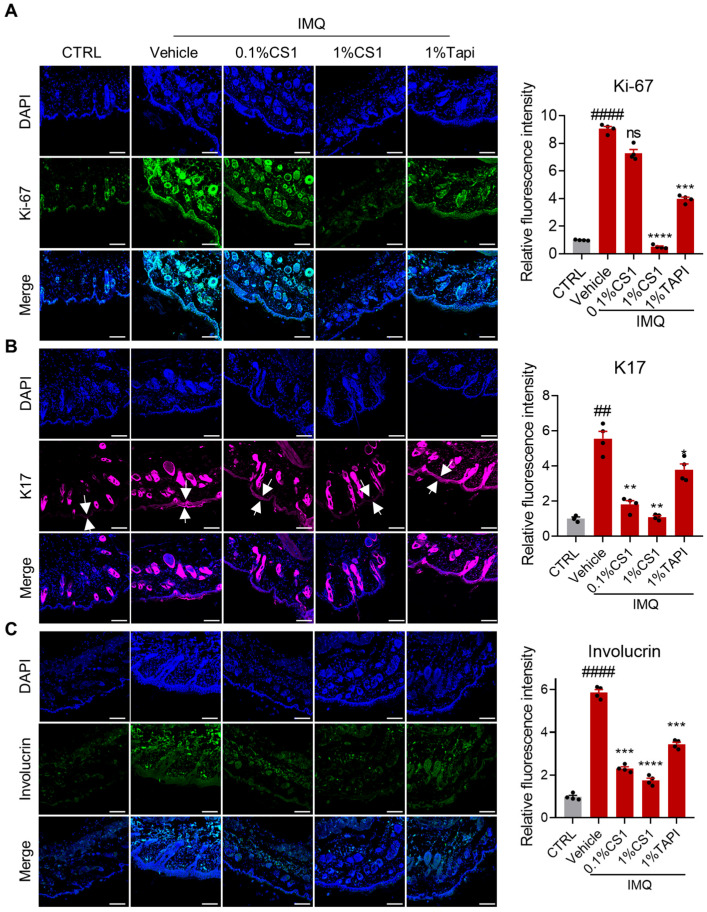
CS1 suppresses the expression of proliferation and differentiation markers in IMQ mice. (**A**–**C**) Immunofluorescence was performed to analyze the expression of KI-67, K17, and p-AKT in skin tissue sections. Scale bar: 100 μm. The values are presented as the mean ± SEM (n = 4 mice skin samples per groups). ## *p* < 0.01 and #### *p* < 0.0001 vs. CTRL. * *p* < 0.05, ** *p* < 0.01, *** *p* < 0.001, and **** *p* < 0.0001 vs. vehicle. *p*-values were calculated using ordinary one-way ANOVA.

The persistent keratinocyte hyperproliferation and altered differentiation are the hallmarks of psoriasis [19]. CS1 effectively inhibited the proliferation of human HACAT keratinocytes in vitro in a dose-dependent manner (Figure S2). Consistently, IMQ markedly induced the expression of the proliferation marker Ki-67, PCNA, and c-Myc in skin lesion, which was significantly inhibited by CS1 treatment (Figure 5A and Figure S3A–C). Moreover, keratin 17 (K17) has been shown to be overexpressed under conditions of hyperproliferative keratinocyte in psoriasis and wound closure [23]. As expected, IMQ significantly enhanced the expression of K17 in skin tissue compared to the control group, while CS1 treatment considerably reduced the level of K17 in skin tissue induced by IMQ (Figure 5B).

Given that involucrin is a late differentiation marker, we next evaluated the effect of CS1 on the level of involucrin in skin tissue of mice treated with IMQ. Notably, IMQ markedly reduced the mRNA level while increasing the protein level of involucrin in skin tissue compared to the control group, and the effect of IMQ on involucrin was reversed by CS1 treatment (Figure 5C and Figure S3E). This result is consistent with the previous studies that the mRNA level of involucrin is downregulated and its protein level is increased in disease models [24,25,26], suggesting a potential post-transcriptional modification accounting for the discrepancy between the mRNA and protein levels of involucrin [27].

### 2.6. CS1 Effectively Inhibits the AKT and MAPK Pathways

**Figure 6:**
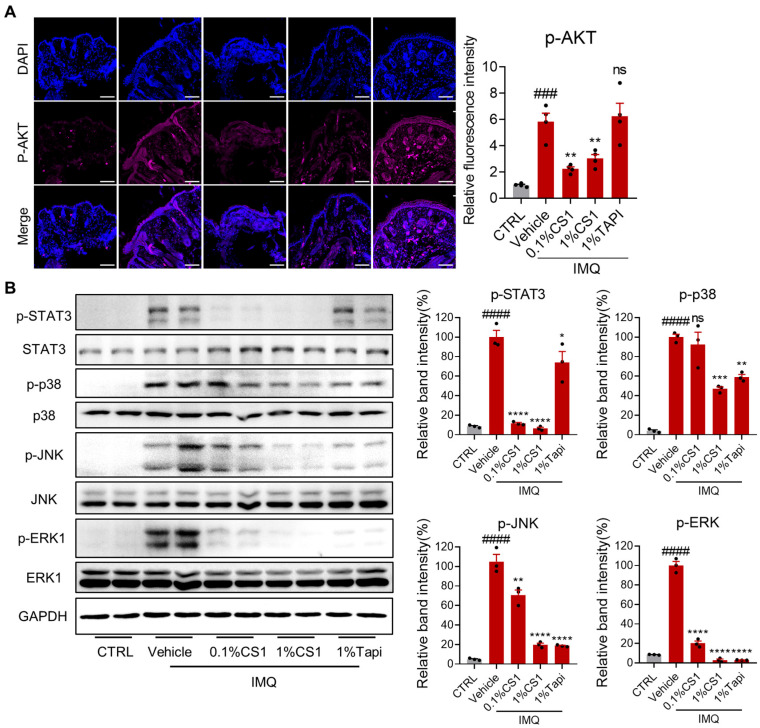
CS1 inhibits the protein expression of the inflammatory signaling cascade. (**A**) Immunofluorescence was performed to analyze the expression of p-AKT in skin tissue sections. (**B**) Immunoblots of p38 and downstream signaling markers with CS1 treatment observed in skin tissues. The values are presented as the mean ± SEM (**A**) n = 4 mice skin samples per groups. (**B**) n = 3 mice skin samples per groups). ### *p* < 0.001 and #### *p* < 0.0001 vs. CTRL; * *p* < 0.5, ** *p* < 0.01, *** *p* < 0.001, and **** *p* < 0.0001 vs. CTRL. *p*-values were calculated using ordinary one-way ANOVA.

The PI3-K/Akt pathway plays a key role in the hyperproliferation of keratinocytes and pathogenesis of psoriasis [28]. Immunohistochemical and Western blotting analysis showed that IMQ markedly induced phosphorylation of Akt in skin lesion compared to the control group, which was effectively inhibited by CS1, but not Tapinarof treatment (Figure 6A). Moreover, the inflammatory MAPK pathway is pivotal to psoriasis pathogenesis [29,30], which is considered as a potential target for the treatment of psoriasis [31]. Given the prominent in vitro and in vivo anti-inflammatory effect of CS1, we next evaluate the effect of CS1 on the activation of the MAPK pathway in skin tissue induced by IMQ. Western immunoblotting analysis showed that IMQ markedly activated the p38/MAPK pathway, as reflected by enhanced phosphorylation of p38, JNK, and ERK1. Both CS1 and Tapinarof treatment significantly reduced phosphorylation of p38, JNK, and ERK1 induced by IMQ. Notably, IMQ also considerably enhanced phosphorylation of STAT3, a key player in the development and pathogenesis of psoriasis and psoriatic-like inflammatory conditions [30,32,33], which was significantly inhibited by CS1, but not Tapinarof treatment (Figure 6B and Figure S4A,B).

### 2.7. CS1 Is a Novel HDAC6 Inhibitor

**Figure 7:**
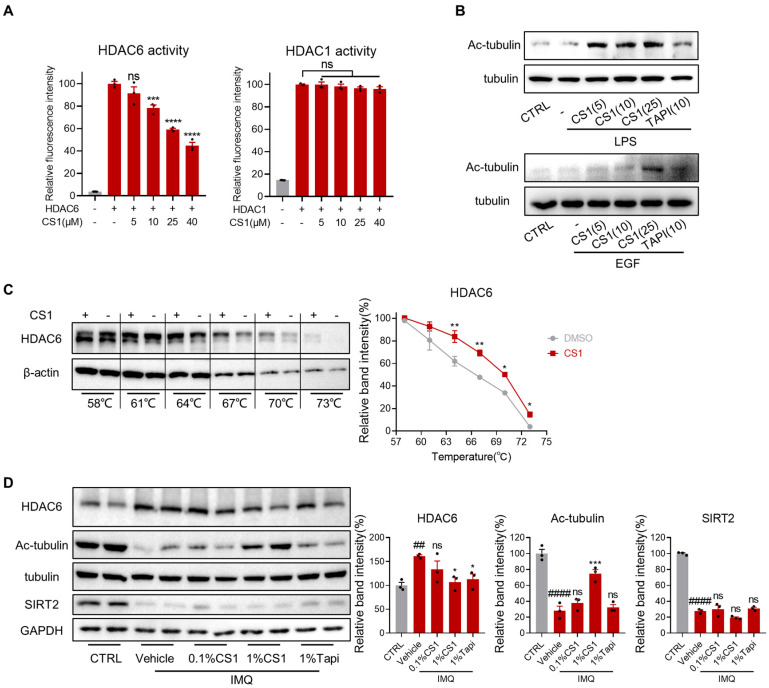
CS1 inhibits the activity of HDAC6. (**A**) Fluorescent probe for HDAC6 and HDAC1 enzyme activity. (**B**) Immunoblots of acetylated tubulin in RAW264.7 cells (left) and HACAT cells (right). (**C**) Cellular thermal shift assay was performed to determine the binding of CS1 and HDAC6. (**D**) Immunoblots of HDAC6 and acetylated-tubulin with CS1 treatment observed in skin tissues. The values are presented as the mean ± SEM (**A**) n = 3 independent samples; (**D**) n = 3 mice skin samples per groups). ## *p* < 0.01 and #### *p* < 0.0001 vs. CTRL; * *p* < 0.5, ** *p* < 0.01, *** *p* < 0.001 and **** *p* < 0.0001 vs. HDAC (**A**) or vehicle (**D**). Immunoblot results in (**B**,**C**) are representative of three independent experiments. *p*-values were calculated using ordinary one-way ANOVA (**A**,**D**) and unpaired two-tailed Student’s *t*-test (**C**).

Given that CS1 effectively inhibited the AKT and MAPK pathways and HDAC6 has been previously shown to regulate both AKT and MAPK pathways, in which HDAC6 associates with AKT and facilitates the activation of AKT [34] or HDAC6 overexpression increased activation of MAPK species including ERK, JNK, and p38 [35], these findings suggest a mechanistic link between CS1 activity and HDAC6 regulation. In addition, several studies indicated that inhibition of HDAC6 alleviates LPS or imiquimod-induced inflammation in macrophages [36,37] and p38MAPK phosphorylation and neuroinflammation in mice [38]. Additionally, HDAC6 inhibition has been shown to ameliorate inflammatory skin conditions such as atopic dermatitis [39]. We thus examined whether CS1 inhibits HDAC6 activity and its downstream signal. We first evaluated the inhibitory effect of CS1 on HDAC6 in vitro using a sensitive substrate probe HDAC-MB [40]. As expected, CS1 effectively inhibited the activity of HDAC6 in a dose-dependent manner while showing no inhibitory effect on the activity of HDAC1 (Figure 7A). Furthermore, Western blotting analysis indicated that CS1 treatment significantly increased the acetylation of a-tubulin, a substrate of HDAC6, in both murine RAW264.7 macrophages and human HACAT keratinocytes in a dose-dependent manner (Figure 7B). To further validate HDAC6 as the intracellular potential protein target of CS1, we used the cellular thermal shift assay (CETSA), which has emerged as a powerful label-free method to assess target engagement for ligands in the native cellular environment [41,42,43]. As shown in Figure 7C, CS1 significantly enhanced the thermal stability of HDAC6 compared to the control DMSO, indicating that there was direct binding between CS1 and HDAC6 in RAW264.7 cells. Notably, IMQ markedly enhanced the protein level of HDAC6 in skin tissue of mice compared to the control group (Figure 7D). Concordantly, the acetylation level of a-tubulin was substantially reduced by IMQ, which was restored by CS1 treatment in a dose-dependent manner, while 1% Tapinarof did not restore the level of acetylated tubulin reduced by IMQ. In contrast, IMQ significantly reduced the protein level of another known tubulin deacetylase SIRT2, which is consistent with the previous study that Sirt2 was downregulated in both IMQ-induced and recombinant mouse IL-23–induced psoriasiform skin tissues [44]. Collectively, these results indicate that CS1 is a novel HDAC6 inhibitor, suggesting HDAC6 as a potential target for the treatment of psoriasis-like inflammation.

## 3. Materials and Methods

### 3.1. Cell Culture

Mouse macrophages RAW 264.7 and the spontaneously immortalized human epidermal keratinocytes HaCaT cells were purchased from Shanghai Cell Bank of the Chinese Academy of Sciences (Shanghai, China). Cells were cultured in Dulbecco’s modified Eagle’s medium (DMEM) (Gibco, Invitrogen Corp., Carlsbad, CA, USA) with 10% fetal bovine serum (ExCell, Taicang, China). All cell lines were incubated in 5% CO_2_ at 37 ℃.

### 3.2. Cell Viability

To analyze the effect of cystamine derivatives on cell proliferation of RAW 264.7 and HaCaT cells, CCK8 (TargetMol, Boston, MA, USA) assay was performed according to the manufacturer’s instructions. Cells were planted in 96-well plates (5000 cells/well), treated with different concentrations of CS1 or Tapinarof and incubated for 24 h. Ten microliters of a CCK8 solution were added into each well and measured spectrophotometrically at 450 nm after 2 h of incubation using a microplate reader (BioTek Synergy H1, Winooski, VT, USA).

### 3.3. Real-Time Quantitative PCR

RAW 264.7 cells were treated with 100 ng/mL LPS and different concentrations of CS1 or Tapinarof for 6 h, and then total RNA was extracted and reverse-transcribed. The products were amplified by real-time PCR. The GAPDH gene served as a reference gene for normalization. Primers of qPCR used in this study can be found in Supplementary Table S2.

### 3.4. Enzyme-Linked Immunosorbent Assay

RAW 264.7 cells were treated with 100 ng/mL LPS and different concentrations of CS1 or Tapinarof for 12 h, and then, the cell supernatants were collected. The levels of cytokines and chemokines were measured using specific ELISA kits for mouse IL-1β (BioLegend, San Diego, CA, USA), TNFα, and IL6 (Invitrogen, Thermo Fisher Scientific, Waltham, MA, USA). Mice skin was collected and analyzed by ELISA kits for mouse IL-1β (BioLegend, San Diego, CA, USA), IL17a, IL23a, TNFα, and IL6 (Invitrogen, Thermo Fisher Scientific, Waltham, MA, USA). The absorbance was measured at 450 nm within 15 min.

### 3.5. IMQ-Induced Psoriasis-Like Skin Inflammation Model

To induce psoriasiform skin lesion, we used IMQ cream (Zhuhai United Laboratories, Zhongshan, China) for continuous topical application to back skin of mice. The 6–8-week female BALB/c mice were randomly divided into 5 groups based on body weight: vehicle, IMQ, 0.1% CS1, 1% CS1, and 1% Tapinarof group; six mice were used in each group, and 30 mice were used totally. Animals were stratified by body weight, and within each stratum, randomization sequences were generated using the RAND function in Microsoft Excel. Mice in all groups, except the vehicle group, received a daily topical application of 62.5 mg IMQ cream on shaved dorsal skin for seven consecutive days. For CS1 topical application, PEG400 was chosen as the matrix for adequate viscosity and ease in application. From day 8 to day 10, compounds were administered to the skin area. The psoriasis area severity index (PASI) was graded according to Supplementary Table S3. Values exceeding ±15% of the mean were excluded from the analysis. All measurements were consistently performed between 8:00 and 12:00 AM to minimize circadian variability, and no standardization was implemented across different operators during data collection. As the phenotypes were group-specific, no blinding was implemented at any stage. All personnel were aware of group allocations. All animal procedures were reviewed and approved by the ethics committee of the Laboratory Animal Center of Peking University Shenzhen Graduate School in accordance with the Policy on the Care, Welfare, and Treatment of Laboratory Animals. The assigned approval or accreditation number is AP0020005.

### 3.6. Histological Analysis

Mouse skin tissues were preserved in 4% paraformaldehyde (Solarbio, Beijing, China) and stored at 4  °C until tissue sectioning. Skin specimens that were embedded in paraffin were cut into consecutive levels of thickness. These sections were then subjected to staining with hematoxylin and eosin (H&E) using established protocols. The purpose of this staining was to assess the presence of epidermal hyperplasia and inflammation in the skin. Following the staining process, the resulting images were captured using a bright-field microscope at magnifications of either 20 × or 40 ×. The images were processed and quantified through scaling or direct counting.

### 3.7. Antibodies

The antibodies used in this study were as follows: FITC anti-F4/80 antibody (11-4801-81, Invitrogen, Thermo Fisher Scientific, Waltham, MA, USA), PE anti- TCR γ/δ antibody (#118108, BioLegend, San Diego, CA, USA), anti-Ki67 antibody (A00254-1, Boster Bio, Wuhan, Hubei, China), anti-pAKT antibody (Proteintech, 66444, Proteintech, Chicago, IL, USA), anti-involucrin antibody (A13311, Abclonal, Wuhan, China), anti-cMyc antibody (67441, Proteintech, Chicago, IL, USA), anti-P38 antibody (AF1111, Beyotime Biotechnology, Shanghai, China), anti-pP38 antibody (AF5887, Beyotime Biotechnology, Shanghai, China), anti-JNK antibody (AF1048, Beyotime Biotechnology, Shanghai, China), anti-pJNK antibody (AF1762, Beyotime Biotechnology, Shanghai, China), anti-ERK antibody (AF1051, Beyotime Biotechnology, Shanghai, China), anti-pERK antibody (AF1891, Beyotime Biotechnology, Shanghai, China), anti-acetylated tubulin (66200, Proteintech, Chicago, IL, USA), anti-α tubulin (11224, Proteintech, Chicago, IL, USA), anti-SIRT2 (A0273, Abclonal, Wuhan, China), anti-HDAC6 (12834, Proteintech, Chicago, IL, USA), anti-GAPDH (#5174, Cell Signaling Technology (CST), Danvers, MA, USA), donkey anti-rabbit Alexa Fluor 488-labeled secondary antibody (ab150073, Abcam plc, Cambridge Biomedical Campus, Cambridge CB2 0AX, UK), iFluor™ 647 conjugated goat anti-rabbit IgG Goat secondary antibody (HA1123, Huabio, Hangzhou, Zhejiang, China), HRP linked anti-rabbit secondary antibody (#7074, Cell Signaling Technology (CST), Danvers, MA, USA), and HRP linked anti-mouse secondary antibody (#7076S, Cell Signaling Technology (CST), Danvers, MA, USA).

### 3.8. Immunofluorescence (IF)

Skin slides were blocked in a solution (10% goat serum, 0.3% Triton) for 1 h at room temperature and incubated with primary antibodies at 4  °C overnight. After washing 3 times in PBS for 10 min each, slices were incubated with secondary antibodies for 1 h at room temperature away from light. The slides were counterstained with DAPI (1:1000, *v/v*) and observed under a fluorescence microscope (Nikon A1R+ confocal microscope; Nikon Instruments Inc., Melville, NY, USA).

### 3.9. Western Blot Analysis

Samples were lysed (RIPA buffer), separated (50 μg/lane by SDS-PAGE) and transferred to PVDF membranes. After blocking (5% milk/TBST, 1 h), membranes were incubated with primary (4 °C, overnight) and secondary antibodies (RT, 2 h). Signals were detected using ECL (EpiZyme, Inc., Shanghai, China).

### 3.10. HDAC6 Activity Assay

HDAC6 activity was measured using a HDAC6 fluorometric probe HDAC-MB as previously described [40]. In brief, the recombinant HDAC6 protein was suspended in the assay buffer and incubated with different concentrations of CS1 for 30 min at room temperature. Subsequently, HDAC-MB was added and incubated for another 30 min. The probe emitted a fluorophore, which was quantified by using a fluorescence plate reader.

### 3.11. HDAC1 Activity Assay

HDAC1 activity was measured using an HDAC1 fluorometric activity assay kit (Enzo Life Sciences, Inc., Farmingdale, NY, USA). The measurements were conducted according to a standardized protocol.

### 3.12. Cellular Thermal Shift Assay

RAW 264.7 cells were pretreated with 25 μM CS1 (1 h), washed and resuspended in PBS. Cells (100 μL aliquots) were heated at different temperatures (3 min), cooled (3 min) and centrifuged (2500 rpm, 5 min, 4 °C). Lysates (RIPA buffer, 30 min on ice) were centrifuged (12,000× *g*, 15 min, 4 °C), and supernatants were analyzed by Western blot.

### 3.13. Wound Healing Assay

Confluent HaCaT cells in 12-well plates were scratched using a pipette tip. After washing, a treated medium was added. Wound closure was monitored at 0, 24, and 48 h and quantified using a microscope system (Olympus CKX31SF; Olympus Corporation, Tokyo, Japan).

### 3.14. Statistical Analysis

Statistical analysis was performed using GraphPad Prism (Version 8.0.1, GraphPad Software, San Diego, CA, USA). Data from ≥ 3 independent experiments are expressed as mean ± SEM. Differences between groups were evaluated by one-way ANOVA with Tukey’s post-hoc test, with *p* < 0.05 considered statistically significant.

## 4. Discussion and Conclusions

Psoriasis is characterized by chronic inflammation induced by infiltration of lymphocytes and neutrophils into the lesion area [45]. Conventional therapies for psoriasis include corticosteroids, vitamin D analogs, calcineurin inhibitors, methotrexate, and cyclosporine, most of which are immunosuppressive agents with potential systemic toxicities [46]. Tapinarof is a first-in-class AhR agonist that was recently approved by the FDA for the topical treatment of adult plaque psoriasis, which is the first non-steroidal topical novel chemical entity launched for psoriasis in more than 25 years, providing an efficacious and well-tolerated therapy in the current management of psoriasis [47,48,49]. In this study, we reported a novel cystamine derivative CS1 that ameliorated imiquimod-induced psoriasis-like inflammation in mice. Mechanistically, CS1 bound and inhibited the deacetylase HDAC6 and subsequently inhibited both the AKT and MAPK pathways. Our findings showed that CS1 was superior to Tapinarof in terms of the efficacy of attenuating IMQ-induced psoriasis-like inflammation and inhibiting hyperproliferation and altered differentiation of keratinocytes, suggesting that HDAC6 may serve as a potential target for drug development in the treatment of psoriasis.

HDAC6, a class IIb histone deacetylase, has been implicated in the pathogenesis of various inflammatory disorders. [50]. HDAC6 overexpression triggers pro-inflammatory responses in macrophages by modulating the ROS-MAPK-NF-κB/AP-1 signaling pathways, leading to a significant increase in the production of pro-inflammatory cytokines like TNF-α, IL-1β, and IL-6 [35]. Specific inhibition of HDAC6 has been shown to alleviate synovial inflammation and protect against joint destruction in collagen-induced or adjuvant-induced arthritis mice [51,52]. Moreover, genetic knockdown or pharmacological inhibition of HDAC6 effectively suppressed atopic dermatitis (AD) by decreasing autophagic flux and cellular features of AD [39]. Selective HDAC6 inhibitors, such as ACY-1215 and Tubacin, ameliorate the development of contact hypersensitivity (CHS) and experimental graft-versus-host disease (GVHD)-like disease through impairment of CD8 T-cell functions [53]. Our findings demonstrated for the first time that pharmacological inhibition of HDAC6 by CS1 effectively attenuated LPS-induced inflammation in vitro and ameliorated IMQ-induced psoriasis-like inflammation in mice, further underscoring the therapeutic potential of HDAC6 inhibitors in inflammatory diseases.

Histone deacetylases (HDACs) have been shown to involve in various diseases and considered as attractive drug targets [54], but the potential of HDAC inhibitors in psoriasis is not fully characterized [55]. Previous studies indicate that HDAC1 is upregulated in psoriatic patients compared to healthy controls [56,57]. Moreover, HDAC3 is proposed to mediate IMQ-induced psoriasis inflammation by interacting with p65, and piperlongumine effectively alleviates psoriasis-like skin inflammation by regulating the interaction between HDAC3 and p65 with IκBα [37]. Currently, several HDAC inhibitors, including Vorinostat, Romidepsin, Panobinostat, and Belinostat, have been approved for the treatment of cancers, but the clinical use of these HDAC inhibitors in other diseases need further investigations [58], and the long-term use of these pan-HDAC inhibitors in psoriasis may be limited by their adverse effects [59]. Given that HDAC6-knockout mice develop normally and reducing endogenous HDAC6 levels restores learning, memory, and a-tubulin acetylation in a mouse model for Alzheimer’s disease (AD), HDAC6 is thus considered as a safer target compared to other HDACs [60,61]. Herein, we demonstrated that HDAC6 was significantly upregulated in skin tissue in the IMQ-induced psoriasis mouse model. CS1 selectively inhibited HDAC6, rather than HDAC1, and effectively alleviated IMQ-induced psoriasis-like inflammation in mice without obvious adverse effect. It should be noted that the selectivity of CS1 against HDAC6 needs more comprehensive testing, and the beneficial effect of other selective HDAC6 should also be tested in psoriatic animal models to validate further the potential of HDAC6 inhibitors in the treatment of psoriasis.

While our study provides novel insights into the immunomodulatory effects of CS1, several limitations should be acknowledged. First, although we demonstrated that CS1 modulates Th17/Th2-type immune responses by reducing IL-17/IL-23 secretion and decreasing γδT cell infiltration at multiple molecular levels, the lack of direct T cell subset analysis (e.g., CD4+ T cells and regulatory T cells) prevents definitive conclusions regarding whether CS1 directly affects T cell differentiation or merely alters cytokine production profiles. Second, the current study did not characterize CS1′s skin absorption kinetics, which would be crucial for understanding its topical pharmacokinetic profile. Third, our cytokine measurements were primarily derived from immune cells (e.g., macrophages), while keratinocyte-derived inflammatory factors were not systematically assessed, leaving potential epithelial-immune crosstalk mechanisms unexplored. These limitations highlight the need for future mechanistic studies employing advanced techniques such as adoptive T cell transfer experiments and single-cell transcriptomics to precisely delineate CS1′s cellular targets and molecular pathways in the immune system.

In conclusion, our study demonstrated that HDAC6 inhibitor, CS1, effectively attenuated LPS-induced inflammation in vitro and ameliorated imiquimod-induced psoriasis-like inflammation in mice. The inhibition of HDAC6 by CS1 markedly inhibited the hyperproliferation of keratinocytes by blocking the AKT pathway and reduced the infiltration of inflammatory cells including macrophages and γδ T cells by inhibiting the inflammatory MAPK and STAT3 pathways. These findings suggest that HDAC6 may serve as a potential target for drug development in the treatment of psoriasis.

## Data Availability

The data used to support the findings of this study are available from the corresponding author on reasonable request.

## Supplementary Materials

The following supporting information can be downloaded at: https://www.mdpi.com/article/10.3390/molecules30153224/s1. The online version contains supplementary materials available at Table S1: List of the structures of the new molecules; Table S2: List of qPCR primer sequences used in this study; Table S3: IMQ mouse model clinical score; Figure S1: CS1 inhibits the proliferation of keratinocytes; Figure S2: CS1 displays no significant toxicity in mice; Figure S3: CS1 induces barrier gene expression in mice; Figure S4: CS1 suppresses MAPK signaling pathway in RAW 264.7 cells.
