# Supplementary material for: A Novel HDAC6 Inhibitor Ameliorates Imiquimod-Induced Psoriasis-Like Inflammation in Mice
Source: Molecules. 2025 Jul 31;30(15):3224. doi: 10.3390/molecules30153224 (PMC12348054; doi:10.3390/molecules30153224)
Supplement: Supplementary file 1 [file molecules-30-03224-s001.zip › molecules-3735150-supplementary.pdf]

Table S1. List of the structures of the new molecules

Name	Structure
**1**	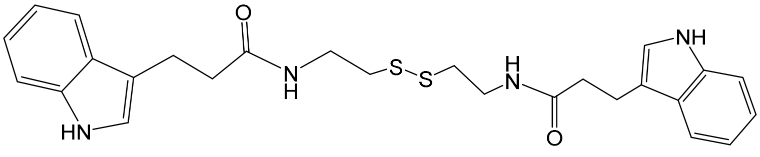 Molecular Weight: 494.67
**2**	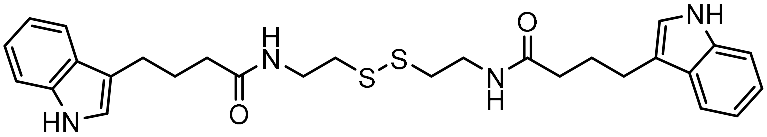 Molecular Weight: 522.7260
**3**	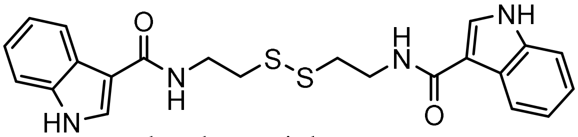 Molecular Weight: 438.5640
**4**	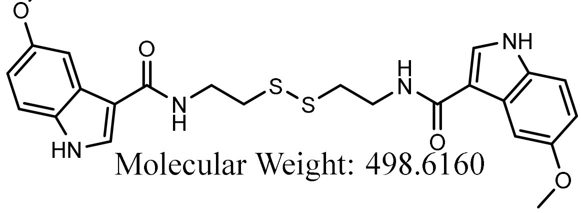 Molecular Weight: 498.6160
**5**	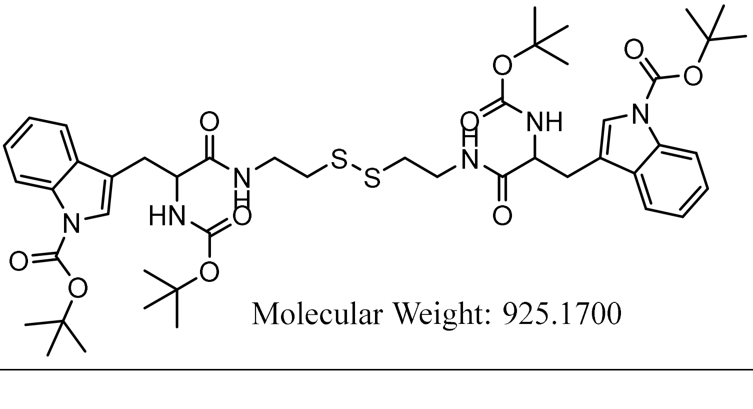 Molecular Weight: 925.1700
**6**	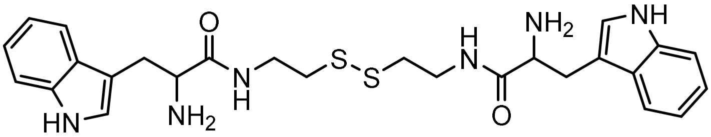 Molecular Weight: 524.7020

**7**	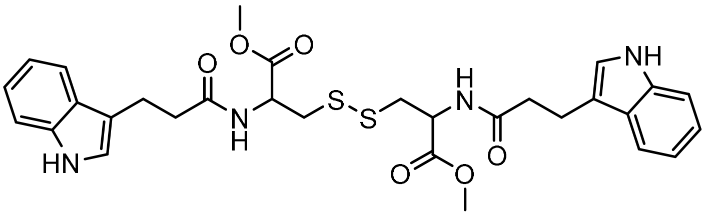 Molecular Weight: 610.7440
**8**	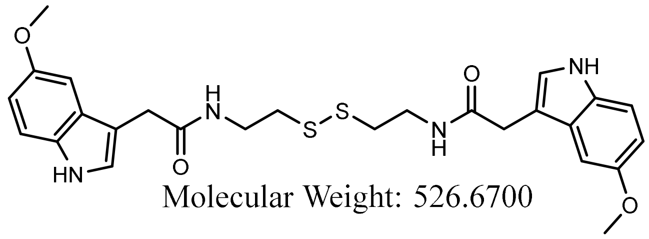 Molecular Weight: 526.6700
**9**	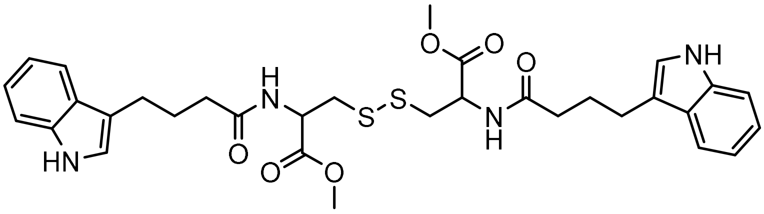 Molecular Weight: 638.80
**10**	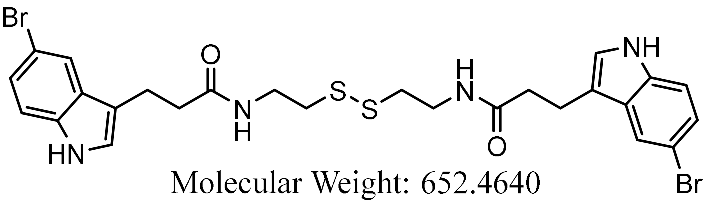 Molecular Weight: 652.4640
**11**	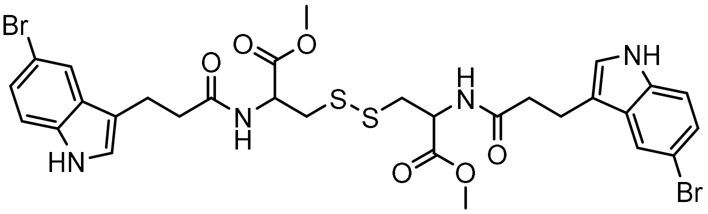 Molecular Weight: 768.5360
**12**	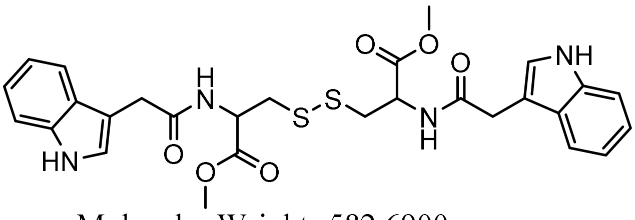 Molecular Weight: 582.6900

**13**	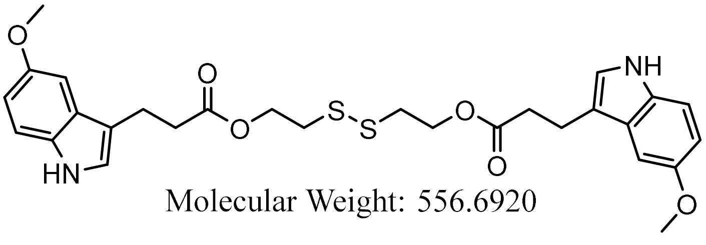 Molecular Weight: 556.6920
**14**	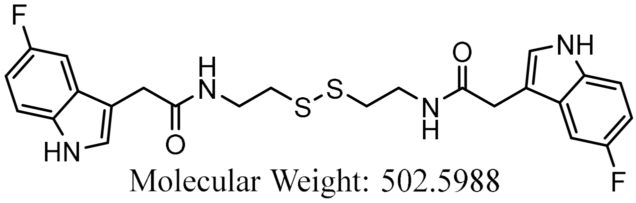 Molecular Weight: 502.5988
**15**	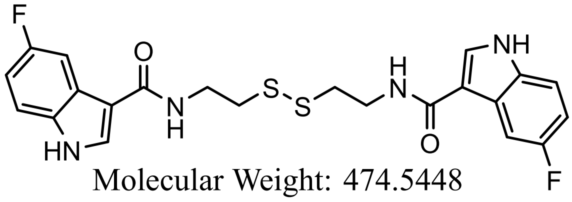 Molecular Weight: 474.5448
**16**	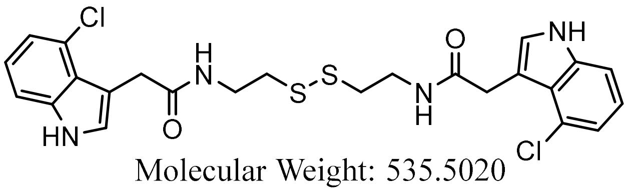 Molecular Weight: 535.5020
**17**	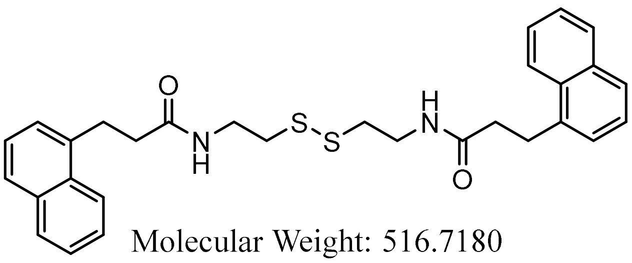 Molecular Weight: 516.7180
**18**	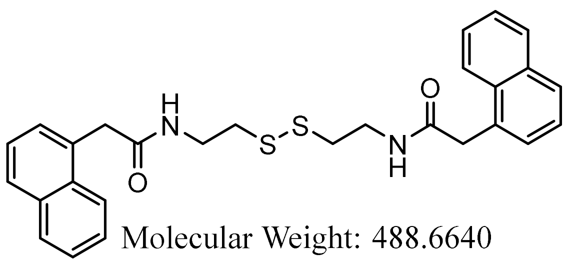 Molecular Weight: 488.6640

Table S2. List of qPCR primer sequences used in this study

Name	Sequence(5'-3')
mIL1β-F	TTCAGGCAGGCAGTATCACTC
mIL1β-R	GAAGGTCCACGGGAAAGACAC
mIL6-F	CTGCAAGAGACTTCCATCCAG
mIL6- R	AGTGGTATAGACAGGTCTGTTGG
miNOS-F	GTTCTCAGCCCAACAATACAAGA
miNOS-R	GTGGACGGGTCGATGTCAC
mCOX2-F	TTCAACACACTCTATCACTGGC
mCOX2-R	AGAAGCGTTTGCGGTACTCAT
mIL18-F	GACTCTTGCGTCAACTTCAAGG
mIL18-R	CAGGCTGTCTTTTGTCAACGA
mIL23a-F	CTCAGGGACAACAGTCAGTTC
mIL23a-R	ACAGGGCTATCAGGGAGCA
mIFNβ-F	CGTGGGAGATGTCCTCAACT
mIFNβ-R	AGATCTCTGCTCGGACCACC
mCXCL10-F	GGTCTGAGTCCTCGCTCAAG
mCXCL10-R	GTCGCACCTCCACATAGCTT
mMCP1-F	TTAAAAACCTGGATCGGAACCAA
mMCP1-R	GCATTAGCTTCAGATTTACGGGT
mTNFα-F	CCCTCACACTCAGATCATCTTCT
mTNFα-R	GCTACGACGTGGGCTACAG
mF4/80-F	CTCAGTCTGCACCAATATCCTG
mF4/80-R	CCACAGAGTTAGAGCAGTTGGAA

mKI-67-F	CAAGGCGAGCCTCAAGAGATA
mKI-67-R	TGTGCTGTTCTACATGCCCTG
mPCNA-F	TTTGAGGCACGCCTGATCC
mPCNA-R	GGAGACGTGAGACGAGTCCAT
mcMYC-F	TTCATCTGCGATCCTGACGAC
mcMYC-R	CACTGAGGGGTCAATGCACTC
mDSC1-F	ACACCTGTAGGCAAAGTGAATC
mDSC1-R	CCTTGACCATCTGAGAGCAAGA
mIVL-F	ATGTCCCATCAACACACACTG
mIVL-R	TGGAGTTGGTTGCTTTGCTTG
mGAPDH-F	AGGTCGGTGTGAACGGATTTG
mGAPDH-R	GGGGTCGTTGATGGCAACA
hcMYC-F	GTCAAGAGGCGAACACACAAC
hcMYC-R	TTGGACGGACAGGATGTATGC
hKI-67-F	GCCTGCTCGACCCTACAGA
hKI-67-R	GCTTGTCAACTGCGGTTGC
hGAPDH-F	GAGTCCACTGGCGTCTTCAC
hGAPDH-R	TCTTGAGGCTGTTGTCATACTTCT

Table S3. IMQ mouse model clinical score

**Psoriasis Score**	**Grade**
Normal	0
Slight erythema	1
Moderate to severe erythema and some plaques	2
Marked erythema and plaques	3
Very marker erythema and plaques	4

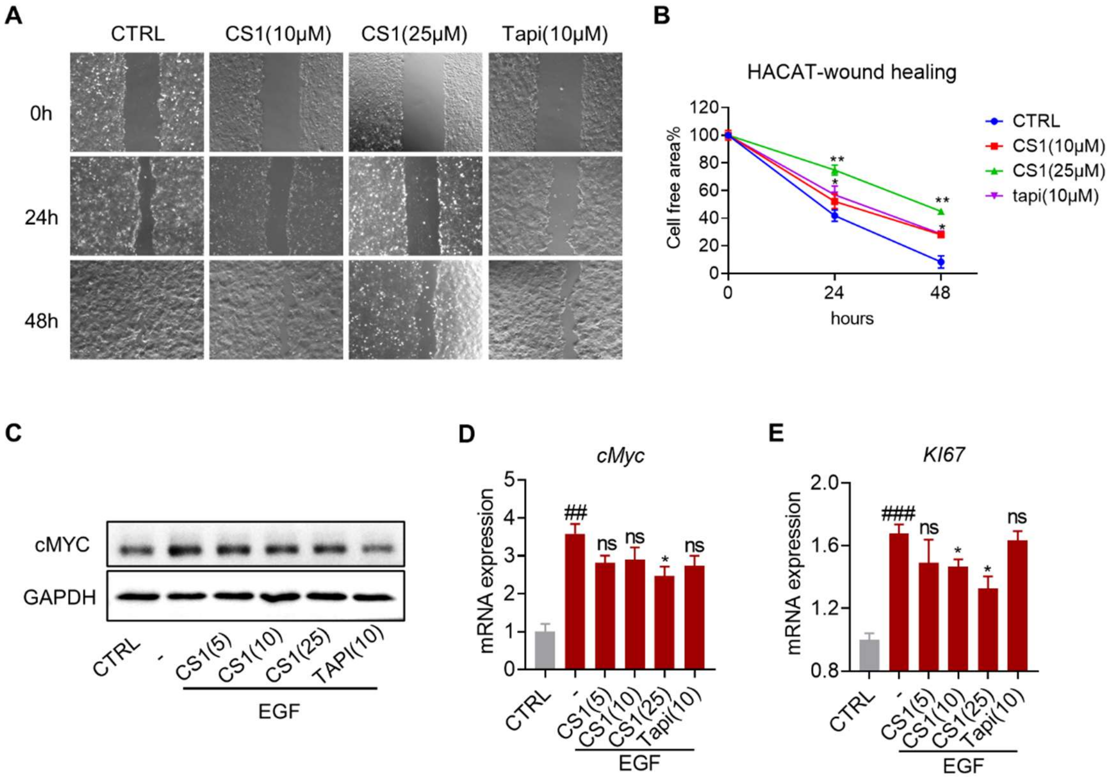

Fig. S1 CS1 inhibits the proliferation of keratinocytes. A Microscopic picture of HaCaT keratinocyte migration assay. **B** Graphical analysis of the effect of CS1 on keratinocyte migration. **C** Western blotting of cMyc protein in HaCaT cells. **D-E** mRNA levels of proliferation related genes in HaCaT cells were examined by qPCR. The values are presented as the means ± SEM. *p < 0.05, **p < 0.01 vs CTRL (**B**) or EGF (**D, E**). ##p < 0.01, ###p < 0.001 vs CTRL, n=6 (One-way ANOVA).

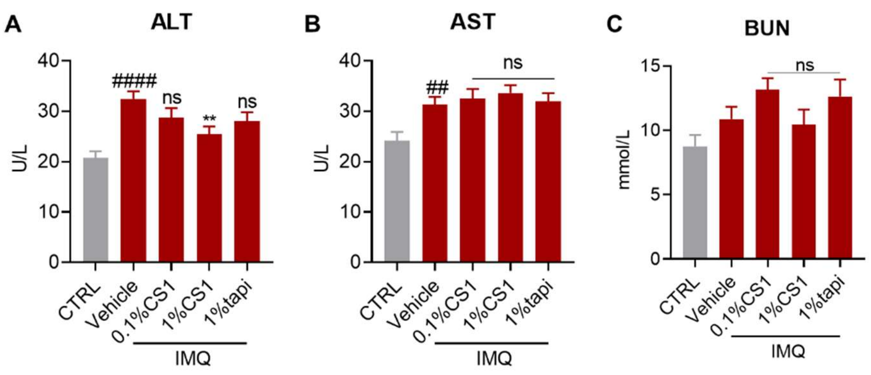

Fig. S2 CS1 displays no significant toxicity in mice. A-C The levels of serum AST, ALT and BUN of mice was measured at the eleventh day, after the sacrifice. The values are presented as the means ± SEM. **$p < 0.01$ vs Vehicle. ##$p < 0.01$, ####$p < 0.0001$ vs CTRL, n=6 (One-way ANOVA).

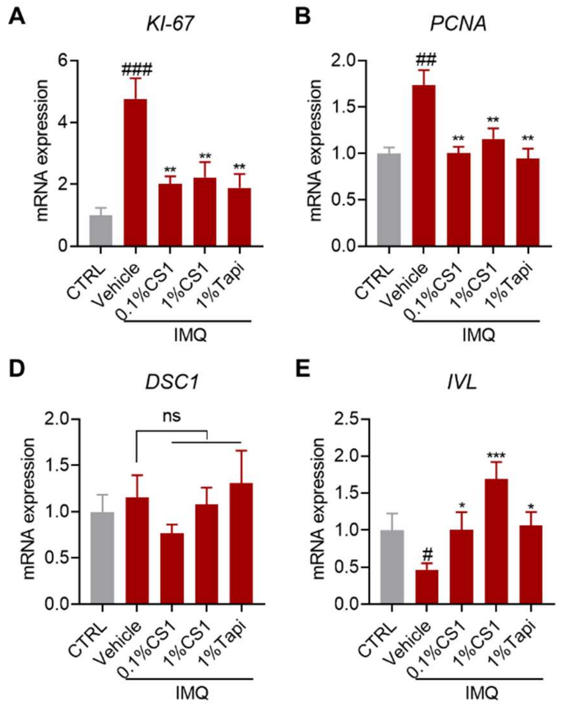

Fig. S3 CS1 induces barrier gene expression in mice. A-C mRNA expression levels of genes related to keratinocyte proliferation. **D-E** mRNA expression levels of genes related to keratinocyte differentiation. The values are presented as the means ± SEM. *p < 0.05, **p < 0.01, ***p < 0.001 vs Vehicle. #p < 0.05, ##p < 0.01, ###p < 0.001 vs CTRL, n=6 (One-way ANOVA).

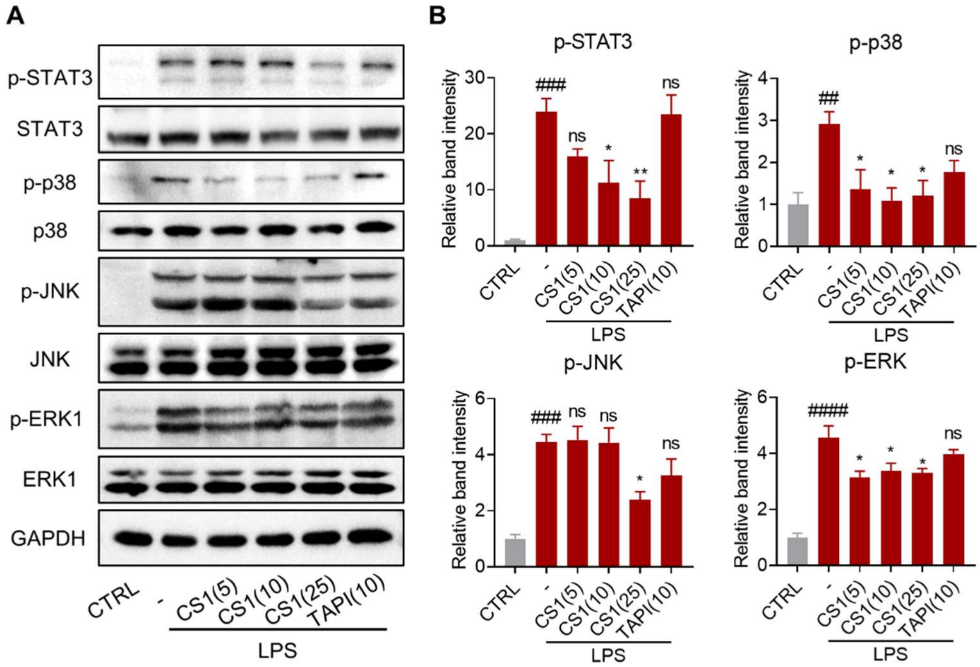

Fig. S4 CS1 suppresses MAPK signaling pathway in RAW 264.7 cells. A Western blotting of MAPK related protein in RAW 264.7 cells. **B** Relative protein levels of Fig. A was quantified. The values are presented as the means ± SEM of three independent experiments. *p < 0.05, **p < 0.01, ***p < 0.001 vs Vehicle. #p < 0.05, ##p < 0.01, ###p < 0.001, ####p < 0.0001 vs CTRL (One-way ANOVA).
